# Wanted—Locality

**Published:** 1892-05-28

**Authors:** 


### Passing Topics.

### WANTED—LOCALITY.

It has been remarked that one disadvantage of living in a country town is that your neighbours easily become more fully acquainted with the events of your domestic life than is agreeable to a retiring disposition. Nothing is too trivial to be noted and little is safe from that exaggeration which renders the most ordinary incidents of your household matters of supreme importance—to your neighbours.

A sensitive mind soon begins to feel the inconveniences entailed by a localisation of existence, and sighs for that independence and freedom from observation and intrusion which are nowhere so easily or so fully obtained as in the centre of a large city. There many live, labour, suffer, and die without becoming the objects of so much as a passing thought on the part of the people next door. Nor is this true only of men and women. In our huge metropolis, institutions are swallowed up and lost sight of almost as readily as individuals. Year by year, with never failing regularity, a great work may go on in our midst, and we are well content to enquire nothing and to know nothing whatever about it. It has never been any business of ours and we have no intention of concerning ourselves with it. That is the exact position accorded to many hospitals and other charitable institutions in relation to the neighbourhood in which they have their habitation. They are in it but not of it, and no one who looks into the matter can fail to arrive at the conclusion that not a few valuable societies whose headquarters are in London suffer considerable disadvantage in this way. So much apparently depends upon personal sympathy and interest, and so little, by comparison, upon a public reputation for good work done.

The value of locality in promoting the prosperity of a charitable organisation has just been pleasantly illustrated in the liberal legacy bequeathed to the Sussex County Hospital by the late Mr. John Silvani, long a resident in Brighton, and this is but one illustration of many. There are metropolitan hospitals which possess localities in the best sense, St. Mary's, for instance, and there are others like St. George's, which although well situated, do not seem to derive from that circumstance the benefit they ought. No doubt it would be easy to confound cause and effect. In the case of hospitals like those we name, which perform an identical work under what appear to be precisely similar conditions, the difference in the treatment they receive at the hands of their neighbours must be due in some measure at least to their own methods. But there are other hospitals of which it is impossible to say that the creation of local sympathy and support rests with themselves.

Locality must always be more or less lacking in the case of special hospitals, and there are general hospitals in poor districts which derive no benefit from its possession. All it brings the latter is an influx of patients, and even patients are known to migrate in a remarkable way to hospitals in districts not their own. In a country town, even of the magnitude of Brighton, attention is more or less focussed upon one chief institution of its class, and *esprit*, so difficult to cultivate in huge cities, is a natural and fruitful product of localised society. Long may provincial institutions find their Silvanis to cherish them. Those that are metropolitan do not begrudge them their good fortune; they only wish they could share in it.

AGRICOLA.

